# *Listeria monocytogenes* Sequence Types 121 and 14 Repeatedly Isolated Within One Year of Sampling in a Rabbit Meat Processing Plant: Persistence and Ecophysiology

**DOI:** 10.3389/fmicb.2018.00596

**Published:** 2018-03-29

**Authors:** Frédérique Pasquali, Federica Palma, Laurent Guillier, Alex Lucchi, Alessandra De Cesare, Gerardo Manfreda

**Affiliations:** ^1^Dipartimento di Scienze e Tecnologie Agro-Alimentari, Alma Mater Studiorum – Università di Bologna, Bologna, Italy; ^2^Laboratoire de Sécurité des Aliments, Agence Nationale de Sécurité Sanitaire de l’Alimentation, de l’Environnement et du Travail, Maisons-Alfort, France

**Keywords:** *Listeria monocytogenes*, the food processing environment, persistence, stress, adaptation

## Abstract

*Listeria monocytogenes* is a foodborne pathogen adapted to survive and persist in multiple environments. Following two previous studies on prevalence and virulence of *L. monocytogenes* ST121 and ST14 repeatedly collected in a the same rabbit-meat processing plant, the research questions of the present study were to: (1) assess persistence of *L. monocytogenes* isolates from the rabbit-plant; (2) select genes associated to physiological adaptation to the food-processing environment; (3) compare presence/absence/truncation of these genes in newly sequenced and publicly available ST121 and ST14 genomes. A total of 273 draft genomes including ST121 and ST14 newly sequenced and publicly available draft genomes were analyzed. Whole-genome Single Nucleotide Polymorfism (wgSNP) analysis was performed separately on the assemblies of ST121 and ST14 draft genomes. SNPs alignments were used to infer phylogeny. A dataset of *L. monocytogenes* ecophysiology genes was built based on a comprehensive literature review. The 94 selected genes were screened on the assemblies of all ST121 and ST14 draft genomes. Significant gene enrichments were evaluated by statistical analyses. A persistent ST14 clone, including 23 out of 27 newly sequenced genomes, was circulating in the rabbit-meat plant along with two not persistent clones. A significant enrichment was observed in ST121 genomes concerning stress survival islet 2 (SSI-2) (alkaline and oxidative stress), *qacH* gene (resistance to benzalkonium chloride), *cadA1C* gene cassette (resistance to 70 mg/l of cadmium chloride) and a truncated version of *actA* gene (biofilm formation). Conversely, ST14 draft genomes were enriched with a full-length version of *actA* gene along with the *Listeria* Genomic Island 2 (LGI 2) including the *ars* operon (arsenic resistance) and the *cadA4C* gene cassette (resistance to 35 mg/l of cadmium chloride). Phenotypic tests confirmed ST121 as a weak biofilm producer in comparison to ST14. In conclusion, ST121 carried the *qacH* gene and was phenotypically resistant to quaternary ammonium compounds. This property might contribute to the high prevalence of ST121 in food processing plants. ST14 showed greater ability to form biofilms, which might contribute to the occasional colonization and persistence on harborage sites where sanitizing procedures are difficult to display.

## Introduction

*Listeria monocytogenes* is a foodborne pathogen adapted to survive in a variety of environmental locations including soil, groundwater, decaying vegetation (Gray et al., 2006). In food-processing plants, *L. monocytogenes* has been repeatedly isolated from both food and the environment. Based on different molecular typing methods, isolates sharing the same profile have been collected over months or years in fish, meat, dairy and vegetable processing plants (Leong et al., 2014; Stasiewicz et al., 2015; Véghová et al., 2017). Strains repeatedly isolated over time in the same plant are considered as persistent. Unfortunately, there is not yet an agreement on specific issues related to the definition of persistence. In particular, the number of times of re-isolation, the sources as well as the period of isolation are not yet uniquely defined (Ferreira et al., 2014).

Persistent strains of high virulence are of major concern since they commonly colonize harborage sites difficult to clean or to reach by sanitizing procedures. These strains typically contaminate different lots of food during several months of production and have been described as responsible of outbreaks including few to hundreds cases spread in time and geographical areas (Tompkin, 2002).

The reasons why *L. monocytogenes* persists in food processing plants is still on debate. One strain can colonize harborage sites by chance (Carpentier and Cerf, 2011). In this view, the persistence of the strain was supposed as more related to characteristics of the environment rather than of the strain itself (Carpentier and Cerf, 2011). Authors failed to identify associations among persistence and particular genes/features of the strain (Ferreira et al., 2011; Stasiewicz et al., 2015). Nevertheless, a differential distribution of specific subtypes in food environments and clinical samples was observed, suggesting that *L. monocytogenes* strains might harbor unique genotypic and phenotypic features facilitating survival and growth and ultimately spread to humans. In foods and food processing environments, *L. monocytogenes* of lineage II and serotype 1/2a has been more frequently collected than lineage I (Orsi et al., 2011). Within serotype 1/2a, Clonal Complex (CC) 121 was the most prevalent clone (17%) (Maury et al., 2016). In particular within CC 121, food source was overrepresented in comparison to clinical one (92.9% vs. 7.0%) (Maury et al., 2016). Human strains belonging to CC121 showed prevalence of 9.5 and 2.3% among 116 strains of the Institute Pasteur *L. monocytogenes* database (Moura et al., 2016) and 262 sporadic cases collected throughout Europe, respectively (Nielsen et al., 2017). Low frequency of CC121 in clinical samples was associated to an attenuated virulence of this subtype, which often carries Premature Stop Codon Mutations in the virulence gene marker *inlA*. (Olier et al., 2002, 2003; Van Stelten et al., 2010; Beier and Bertilsson, 2013; Palma et al., 2017).

The CC14 is another clonal complex of serotype 1/2a. Compared to CC121, CC14 was seldom isolated in foods in France (1.4%) (Maury et al., 2016). Similarly, low detection values were reported in 19 meat processing plants located in Northern Italy (5.7% over 69 tested isolates) in comparison to CC121 (23%) (Morganti et al., 2016). Besides a low prevalence in foods, CC14 was described to gather a high percentage of isolates of clinical source (29.5%) (Maury et al., 2016). Within CC14, hypervirulent strains were described. In particular, a Multi Locus Sequence Type (ST) 14 strain was associated to a case of invasive listeriosis. Further molecular analyses revealed that this strain belongs to epidemic clone (EC) III (corresponding to Multi-Virulence Locus Type (VT) 1) previously linked to a sporadic case as well as a multi-state outbreak occurred in United States in 1988 and 2000, respectively (Kathariou, 2002; Mammina et al., 2013).

Physiological adaptation (ecophysiology) to environmental stresses including resistance to antimicrobials, heavy metals and quaternary ammonium compounds (QAC) as well as adaptation to cold, salt, acid, oxidative stresses, desiccation and ability of biofilm formation are often described in *L. monocytogenes* isolated from food processing plants (Lebrun et al., 1994; Kathariou, 2002; Srinivasan et al., 2005; Mullapudi et al., 2008; Ryan et al., 2010; Hein et al., 2011; Ratani et al., 2012; Müller et al., 2013; Ortiz et al., 2015; Kovacevic et al., 2016; Xu et al., 2016; Harter et al., 2017; Hingston et al., 2017). Studies on ST121 strains have been recently performed aiming at identifying genes associated to persistence and/or adaptation to food-processing environmental stresses. In particular, deletions of *lmo02774-lmo2776* were described along with no significant association of genes (Holch et al., 2013; Knudsen et al., 2017). In one study, ST121 isolates were found to harbor the *qacH* carrying transposon Tn*1688* (QAC resistance) (Ortiz et al., 2015). No similar studies are available on ST14.

Analyses based on Whole Genome Sequencing (WGS) have recently revealed an unprecedented potential for multiple investigations as a one-serve-all approach. WGS-based typing showed valuable potential in pathogen fingerprinting. In particular, a high discrimination power has been achieved by single nucleotide polymorphisms (SNPs) analysis. This is particularly relevant to discriminate strains showing high genetic similarity as determined by same Pulse Field Gel Electrophoresis (PFGE) or 7-loci MLST, as it is the case during a persistent event (Ferreira et al., 2014).

Based on WGS-data, studies on presence/absence/truncation of a wide number of genes can be performed at once. Genes described as strongly associated to a specific phenotype can be selected through a literature search, and traced on sequenced genomes for prediction of that specific phenotype. At present, freely available tools unable a quick screening of genomes for thousands of genes selected for their association to virulence or antimicrobial resistance (McArthur et al., 2013; Zankari et al., 2012). Genes, useful to predict phenotypic traits associated to *L. monocytogenes* physiological adaptation to environmental conditions, have not been collected so far in a unique dataset.

In a previous study on prevalence of *L. monocytogenes* in four Italian rabbit meat processing plants, isolates indistinguishable by 7-loci MLST, Multi Locus Variable number tandem repeats Analysis (MLVA) type and *Apa*I-PFGE were repeatedly collected over time. Isolates were sampled from carcasses, meat cuts, meat products and the meat-processing environment (De Cesare et al., 2017). The dataset of this study was used to investigate further the potential virulence of specific subtypes. For this purpose, in a second study a specific focus was addressed on two subtypes gathering isolates of 7-loci MLST ST121 and ST14 collected over 1 year in the same processing plant (Palma et al., 2017). In this latter study, ST14 isolates showed higher virulence potential than ST121 as suggested by *in silico* virulotyping (Palma et al., 2017). In particular, all ST14 isolates belonged to VT107, which differed from ECIII (VT1) for only four nucleotides (Murugesan et al., 2015). Moreover, all ST121 and no ST14 genomes carried a truncated version of the *actA* gene and an *inlA* gene with a premature stop codon of type 6, both described as associated to attenuated virulence (Smith and Portnoy, 1997; Olier et al., 2002, 2003; Van Stelten et al., 2010).

In the present study, the ST121 and ST14 *L. monocytogenes* isolates, collected over 1 year on the same rabbit meat processing plant, were studied with a specific focus on persistence and physiological adaptation to food-processing environmental stresses. In particular, the research questions were: (1) assess persistence of *L. monocytogenes* isolates from the rabbit-plant; (2) select genes associated to physiological adaptation to the food-processing environment; (3) compare presence/absence/truncation of selected genes in newly sequenced and publicly available ST121 and ST14 genomes. A particular focus has been placed on the evaluation of putative gene enrichment in the two subtypes as well as identification of putative markers of ecophysiology associated to survival and growth of rare subtype ST14 of *L. monocytogenes*.

## Materials and Methods

### *Listeria monocytogenes* Isolates From Italian Meat Processing Plants, Sequencing and *de Novo* Assembly

*Listeria monocytogenes* isolates included in the present study belong to a subset of isolates collected from November 2005 to November 2006 within a previous study on prevalence of *L. monocytogenes* in rabbit meat-processing plants (De Cesare et al., 2017). This subset included isolates belonging to two subtypes and collected from plant A. Isolates were considered as potentially persistent when they belonged to the same subtype and they were collected more than six times over a period of more than 6 months from different sources (rabbit carcasses, rabbit meat cuts, rabbit meat products, and the food processing environment). Each subtype gathered isolates indistinguishable by serotype, *Apa*I-PFGE, 7-loci MLST, MLVA and automated ribotype (De Cesare et al., 2017). The genomes of this subset of *L. monocytogenes* isolates were sequenced and *de novo* assembled in a previous study (Palma et al., 2017). Briefly, the genomes of 33 *L. monocytogenes* isolates belonging to ST121 (6) and ST14 (27) were previously sequenced by MiSeq (Illumina) platform. Paired-end reads were *de novo* assembled using the INNUca pipeline^1^, which consists of several modules (e.g., Trimmomatic, SPAdes, Pilon) and QA/QC steps. Information on quality parameters of *de novo* assemblies were included in the previous study (Palma et al., 2017).

### Publicly Available *L. monocytogenes* Genomes Included in the Present Study

A selection of draft genomes from ENA was carried out in order to: (1) explore the genetic distances of newly sequenced *L. monocytogenes* genomes in comparison to publicly available genomes; (2) study ST-related genetic markers associated to ecophysiology. In particular, 196 ST121 and 44 ST14 publicly available draft genomes were included. These genomes were from strains isolated from both food processing environments and humans. Moreover, they were widely distributed over time and geographical locations (**Supplementary Table S2**).

### Whole-Genome SNP Analysis

Single nucleotide polymorphisms (SNPs) calling was performed using the Snippy v2.6 pipeline^2^. *De novo* assemblies of ST121 and ST14 genomes were analyzed separately. A newly generated draft genome for each *L. monocytogenes* ST was chosen as reference genome: LSALM51 for ST121 and LSALM1 for ST14. After removal of Illumina Nextera adapters and low-quality sequences (Phred scores of <10), the *de novo* assemblies were mapped to the reference genome with the Burrows-Wheeler Aligner (BWA) v0.7.12 using default parameters (Li and Durbin, 2009). After mapping, average depths were determined with SAMtools v1.3 (Li et al., 2009). Variants were called using Freebayes v0.9.20 (Garrison and Marth, 2012) with the following parameters: minimum base quality of 20, minimum read coverage of 10X, and 90% read concordance at a locus. Snippy was used to pool all SNP positions called in at least one isolate and investigate all isolates. Alignments of all SNPs were produced in Snippy and used to infer a high-resolution phylogeny. A maximum likelihood (ML) tree was constructed using the PhyML v2.4.4 program to analyze the SNP differences between isolates and FigTree v1.4.3 software^3^ was used to visualize the tree rooting at midpoint for each ST. Draft genomes were tentatively considered as belonging to the same persistent clone if the following criteria were fulfilled: (1) the difference between draft genomes and the reference genome was equal or lower than 25 SNPs; (2) draft genomes belonged to isolates collected from different origins (food and the processing environment) for at least six times during a time frame of at least 6 months. The cut-off of 25 SNPs was used as previously suggested (Nielsen et al., 2017).

### Dataset of Putative Gene Markers of Ecophysiology in *L. monocytogenes*

An extensive literature review was conducted on PubMed^4^ using following keywords: “antimicrobials,” “QAC,” “heavy metals,” “bacteriocins,” “cold,” “high salt concentration,” “low pH,” “desiccation,” “blue-light,” “biofilm,” “*L. Monocytogenes*,” and “genes.” At the time of analysis (October 2017), the system retrieved overall 664 peer-reviewed published papers. The abstract of each of these papers was read with the purpose to identify genes associated to the response of *L. monocytogenes* to each stress. Around 100 papers were selected based on the abstract. These papers were thoroughly read in order to identify and specifically select genes with a strong association to the related phenotype. For this purpose, only genes confirmed by insertional mutagenesis or mutant selection experiments were included (**Supplementary Table S1**). All GenBank accession numbers and related sequences reported in published papers were checked by nucleotide BLAST^5^ and CLUSTAL Omega^6^ for alignment. For each gene, the following informations were reported: name of the gene, locus tag, annotation, main function, localization, reference paper, GenBank accession number and direct link to the web page of the sequence in NCBI^7^ (**Supplementary Table S1**).

### Screening of Genes of Ecophysiology

A multifasta file was generated with the sequences of all selected genes (**Supplementary Table S1**). Nucleotide BLAST was run locally using ABRricate pipeline^8^ on 202 ST121 as well as 71 ST14 draft genomes (**Supplementary Table S2**) with the mutifasta file as gene database. Based on the output matrix of gene presence/absence, a heatmap was built using morpheus software^9^. Morever, results were analyzed for statistically significant differences at 95% confidence by *t*-test.

### Antimicrobial Susceptibility

For phenotypic confirmation, newly sequenced strains carrying antimicrobial resistant genes (*ampC, tetA*) were tested for susceptibility against ampicillin and tetracycline by disk diffusion method (Clinical and Laboratory Standards Institute [CLSI], 2010; Jamali et al., 2015). The assay was performed on Mueller Hinton Agar plates supplemented with 5% defibrinated sheep blood (Thermo Scientific, Milan, Italy). Ampicillin (10 μg) and tetracycline (30 μg), were applied as antibiotic agents following Clinical and Laboratory Standards Institute [CLSI] recommendations for fastidious organisms (document M45-A2).

### Cadmium Chloride and Benzalkonium Chloride Susceptibility

Determination of cadmium chloride and benzalkonium chloride resistance was performed as previously described (Mullapudi et al., 2008). Briefly, a single colony from a blood agar plate culture was suspended in 100 μl of tryptic soy broth (Thermo Fisher Scientific, Milan, Italy). Three microliters of the suspension were spotted in duplicate onto: (1) Mueller Hinton Agar Cation adjusted (MHBII) containing 2% defibrinated sheep blood (Thermo Fisher Scientific) (control); (2) MHBII (Thermo Fisher Scientific) containing 2% defibrinated sheep blood (Thermo Fisher Scientific) and 70 mg/l of cadmium chloride anhydrous (Sigma, Milan, Italy); (3) MHBII (Thermo Fisher Scientific) containing 2% defibrinated sheep blood (Thermo Fisher Scientific) and 35 mg/l of cadmium chloride anhydrous (Sigma, Milan, Italy); (4) Mueller Hinton Agar (MHB) (Thermo Fisher Scientific) containing 2% defibrinated sheep blood (Thermo Fisher Scientific) and 10 mg/l benzalkonium chloride (Sigma). Positive and negative control strains were included. All plates were incubated at 37°C for 48 h.

### Crystal Violet Staining Assay

In order to test the ability of biofilm formation, representative isolates of identified clones of ST121 and ST14 isolates of *L. monocytogenes* were submitted to the crystal violet staining assay as previously described (Stepanović et al., 2004). Briefly, 20 μl of an overnight bacterial culture were added to 230 μl of Brain Heart Infusion broth (BHI, Thermo Scientific, Milan, Italy) into each well of a sterile 96-well not tissue treated polystyrene microplate (Sarstedt, Milan Italy). Plates were incubated at 35°C for 24 h. The content of each plate was discarded and 300 μl of sterile distilled water were added to each well. This washing step was repeated three times. Adherent bacteria were fixed with 250 μl of methanol per well. After 15 min, methanol was discarded and plates air-dried overnight. Biofilms were stained with 250 μl per well of Crystal violet (Gram-color staining set for microscopy; Merck) for 5 min. Excess stain was rinsed off and microplates air-dried. Then, attached bacteria were solubilized with 250 μl of 33% (v/v) glacial acetic acid per well. Finally, the optical density (OD) of each well was measured at 570 nm using Infinite^®^ F50 Absorbance Microplate Reader (Tecan Group Ltd, Männedorf, Switzerland). According to Stepanović et al. (2004), based on the OD produced by bacterial films, strains were classified as no, weak, moderate or strong biofilm producers. All isolates were tested in triplicate.

### Nucleotide Sequence Accession Numbers

*De novo* assembled genomes of the 33 *L. monocytogenes* isolates included in this study were submitted to GenBank under BioProject no. PRJNA396103 with individual BioSample identification (ID) numbers SAMN07420940 to SAMN07420973.

## Results

### Whole-Genome SNP Analysis

**FIGURE 1:**
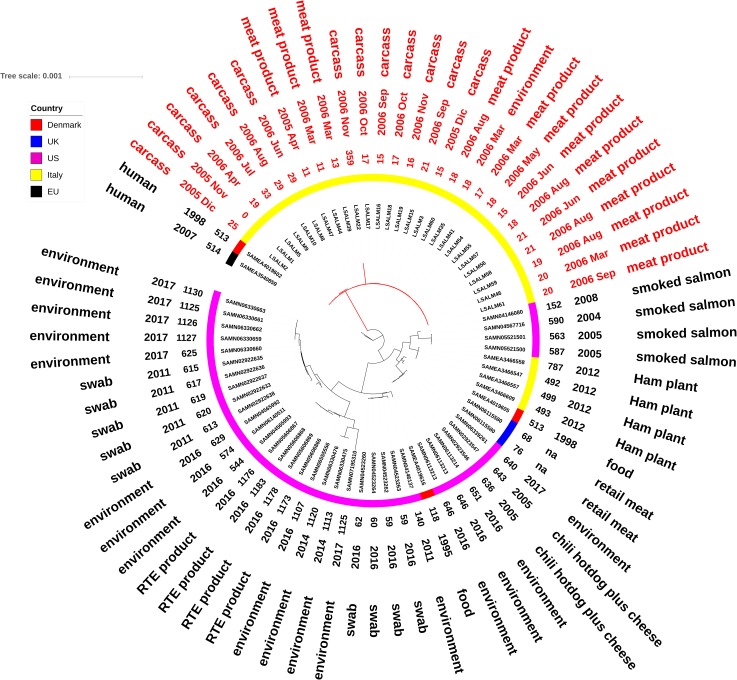
Maximum likelihood tree showing genetic distances between ST14 *L. monocytogenes* draft genomes based on whole-genome core SNPs. The tree tips list study ID of the isolate, date of isolation, origin (rabbit carcass, rabbit meat cut, rabbit meat product, the food processing environment) and the SNP count of the ID genome in comparison to the reference (LSALM1). The scale bar refers to the branch length representing the number of nucleotide substitutions per site.

**FIGURE 2:**
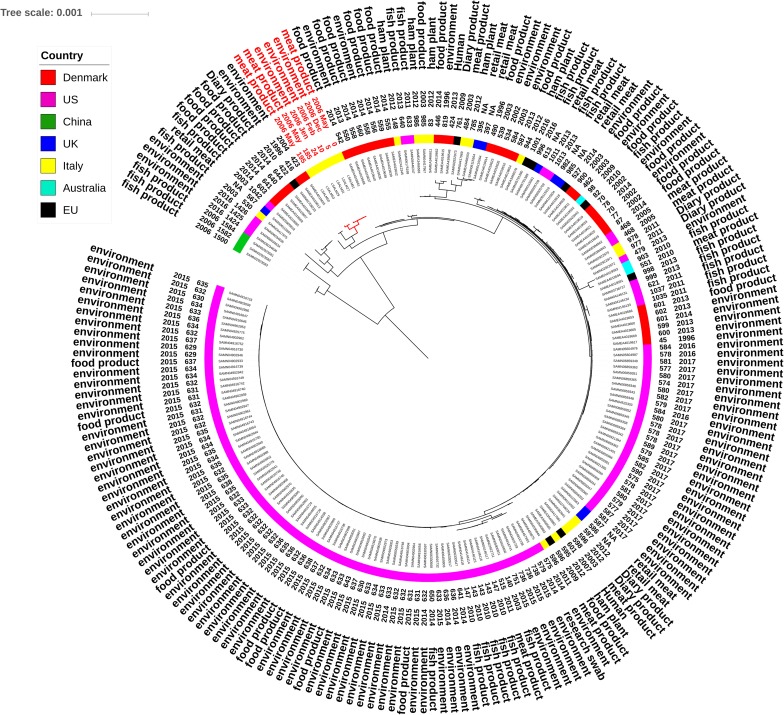
Maximum likelihood tree showing genetic distances between ST121 *L. monocytogenes* draft genomes based on whole-genome core SNPs. The tree tips list study ID of the isolate, date of isolation, origin (rabbit meat cut, the food processing environment) and the SNP count of the ID genome in comparison to the reference (LSALM51). The scale bar refers to the branch length representing the number of nucleotide substitutions per site.

In order to increase the resolution of whole-genome SNP analysis, assemblies of ST121 and ST14 sequenced isolates were mapped separately against *de novo* assemblies of the reference genomes (i.e., LSALM51, LSALM1 strains). The resulting ML trees are reported in **Figures 1**, **2**. Within ST14, 23 out of 27 isolates collected from the rabbit meat-processing environment as well as from rabbit meat carcasses, meat cuts and products from November 2005 to November 2006, shared SNPs counts ranging from 0 to 25 SNPs in comparison to the reference genome LSALM1 and were considered as belonging to the same persistent clone. Three isolates (LSALM8, LSALM9, LSALM10), collected from rabbit carcasses from June to August 2006, showed 29–33 SNPs whereas one isolate (LSALM22), collected in November 2006 showed 359 SNPs (**Figure 1**).

ST121 isolates were clearly differentiated in two distinct clusters (**Figure 2**). One cluster included isolates LSALM50 and LSALM53, collected from rabbit meat cuts in May 2006 and sharing 204 and 214 SNPs in comparison to the reference genome LSALM51. The second cluster included the reference along with LSALM27, LSALM29, LSALM31. These isolates, sharing from 0 to 17 SNPs, were collected from the rabbit meat-processing environment as well as from meat cuts from December 2005 to May 2006 (**Figure 2**).

Public ST121 draft genomes showed genetic distances ranging from 45 SNPs (processing environment, Danmark) to 1584 SNPs (fish product, China) in comparison to the internal reference sequence LSALM51 (**Figure 1**). Public ST14 draft genomes displayed SNPs differences ranging from 60 SNPs (environmental swab, United States) to 1183 SNPs (RTE product, United States). These results confirm the high clonality of *L. monocytogenes* especially within the same ST.

### Dataset of Putative Gene Markers of Ecophysiology in *L. monocytogenes*

Overall 94 genes, described in 41 published papers, were included in the dataset (**Supplementary Table S1**). All selected genes were identified as strongly associated to specific phenotypes related to physiological adaptation of *L. monocytogenes* to environmental stresses encountered in food processing plants. In particular the genes were associated to: resistance to antimicrobials, QAC, heavy metals and bacteriocins; adaptation to cold, high salt concentration, low pH, desiccation and biofilm formation (**Supplementary Table S1**).

Fourteen genes were included related to resistance to different antimicrobial classes such as tetracycline, ampicillin, vancomycin, streptomycin, chloramphenicol/florfenicol, sulphonamides, erythromycin, and fluoroquinolones (Poyart-Salmeron et al., 1992; Charpentier et al., 1993; Roberts et al., 1996; Godreuil et al., 2003; Srinivasan et al., 2005; Lungu et al., 2011; Jamali et al., 2015).

Seven genes associated to resistance to benzalkonium chloride were included (*qacH, qacA, qacC, bcrA, bcrB, bcrC*, and *emrE*). These genes are associated to the active efflux pump of QAC (Elhanafi et al., 2010; Müller et al., 2013; Xu et al., 2014; Kovacevic et al., 2016; Nielsen et al., 2017).

Sixteen genes associated to resistance to cadmium and arsenic were included. In particular, three gene cassettes, *cadA1C*, *cadA2C*, and *cadA3C* were associated to resistance to 70 mg/l of cadmium chloride, whereas *cadA4C* was associated to resistance to 35 mg/l (Mullapudi et al., 2010; Parsons et al., 2017). Regarding resistance to arsenic, *ars* genes were associated to resistance to 500 mg/l of sodium (meta) arsenite (Lee et al., 2013).

Regarding bacteriocins resistance, 5 genes associated to the response of *L. monocytogenes* to cell-envelope stress were included: *virR*, *virS*, *mprF*, *lia*R, *anrB* (Thedieck et al., 2006; Collins et al., 2010; Bergholz et al., 2013; Kang et al., 2015).

As far as stress adaptation is considered, different mutant selection experiments demonstrated the important role of the *sigB* gene (sigma factor B) in the regulation of expression of several genes associated to adaptation to environmental stresses. In particular the knockout of *sigB* was directly associated to adaptation to desiccation in *L. monocytogenes* (Huang et al., 2015).

As far as blue-light is considered, the gene *lmo079*9, coding for a blue-light receptor, was strongly associated to the adaptation of *L. monocytogenes* to this particular stress (Ondrusch and Kreft, 2011; O’Donoghue et al., 2016).

*Listeria monocytogenes* can adapt to cold following different pathways. One pathway includes cold shock proteins cspB and cspD (Schmid et al., 2009). Another pathway includes the glycine/betaine transporter system which mediates the uptake of osmolytes, such as glycine, betaine and carnitine, important for adaptation to both cold and high salt concentration (Angelidis and Smith, 2003). Overall, 13 genes related to adaptation of *L. monocytogenes* to cold and/or high salt concentration were included in the dataset (Angelidis and Smith, 2003; Schmid et al., 2009; Markkula et al., 2012; Pöntinen et al., 2015).

The Stress Survival Islet 1, corresponding to a cassette of five genes (lmo0444-lmo0448), was associated to the survival and growth of *L. monocytogenes* under suboptimal conditions. In particular, the knockout of the entire SSI-1 was associated to an impaired ability of this food-borne pathogen to grow at low pH and high salt concentrations (Ryan et al., 2010). Within this gene cassette, *gadD1* (lmo0447) and *gadT1* (lmo0448) encode for a glutamate decarboxylase and an amino acid transporter both described as specifically involved in the adaptation to low pH (Feehily et al., 2014).

Along the GAD System, the ADI system might be activated in response to low pH. The *arc* gene is involved in transformation of arginine into ornithine with ammonia as by product, which increases the pH. The ADI system has been described in response to mild acid pH (Feehily et al., 2014). Overall, 16 genes associated to low pH adaptation were included in the dataset (Abram et al., 2008; Ryan et al., 2009; Ryan et al., 2010; Feehily et al., 2014).

The Stress Survival Islet 2 (SSI-2) was firstly described as a cassette of two genes of *Listeria innocua* often present in place of SSI-1 in *L. monocytogenes* ST121 (Hein et al., 2011). SSI-2 was more recently associated to alkaline and oxidative stress in *L. monocytogenes* (Harter et al., 2017).

Regarding adaptation to desiccation, seven genes related to motility of *L. monocytogenes*, were recently associated to this specific phenotype (Hingston et al., 2015). In particular, these genes were found to be downregulated in desiccation tolerant *L. monocytogenes*.

As far as the ability of biofilm is concerned, 8 genes associated to biofilm formation were included in the dataset. Biofilm formation is essential for survival of *L. monocytogenes* and further contributes to bacterial persistence in the food processing environment (Popowska et al., 2017).

### Screening of Putative Gene Markers of Ecophysiology and Related Phenotypic Tests

**FIGURE 3:**
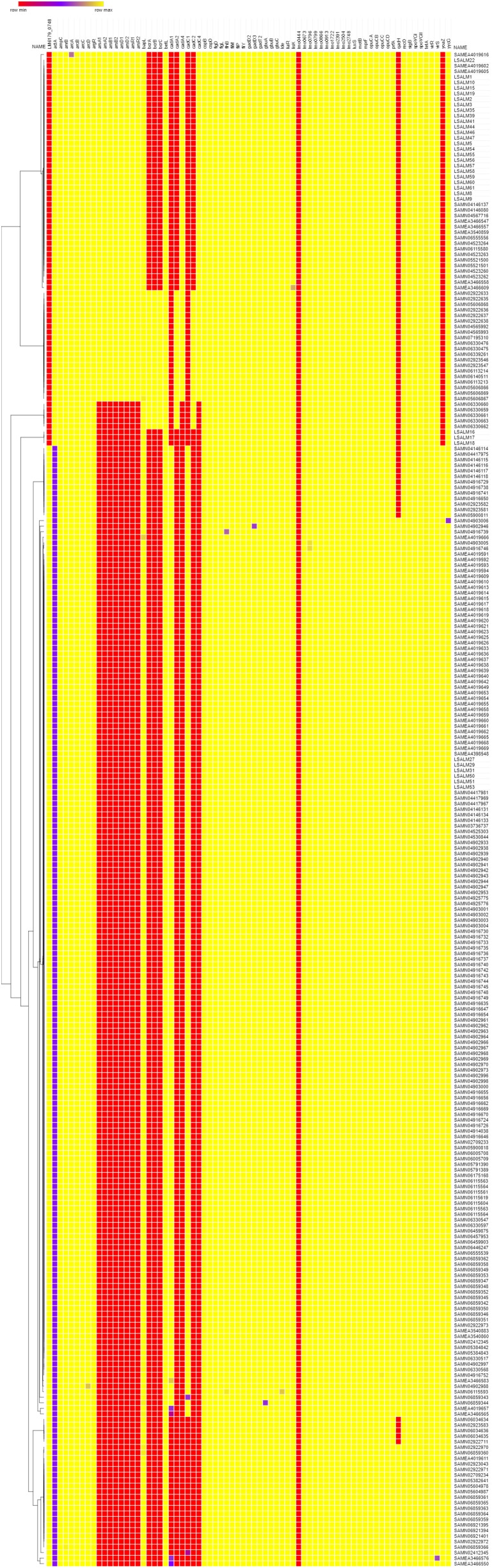
Heatmap of the 202 ST121 and 71 ST 14 genomes function to the ecophysiology gene dataset. The rows represent the genomes included while columns represent each gene of the dataset. The colors represent the percentage of identity and length of genes. Red represents lowest percentage while yellow highest percentage. Genes not found in any of the tested genomes were not included.

The 94 putative gene markers of ecophysiology were screened on 202 and 71 genomes of *L. monocytogenes* ST121 and ST14, respectively (**Figure 3**). Twenty-one genes were not found in any of the tested draft genomes and were not included into the heatmap (**Figure 3**).

As far as antimicrobial resistance gene markers are concerned, all genomes were positive for *ampC* and *tetA* associated to penicillin and tetracycline resistance, respectively. However, phenotypic tests did not confirm genetic results. Although disk diffusion breakpoints are not available for *Listeria*, all isolates showed zone diameters equal or higher than 30 mm to tetracycline and ampicillin, suggesting susceptibility (data not shown). Further analysis should be performed in order to assess the reason behind these discordant results.

Regarding resistance to QAC, 25 ST14 genomes (35.2%) and 184 (91.2%) ST121 genomes were positive for the *bcrABC* locus and the *qacH* gene, respectively. All ST14 genomes but 8 (88.7%) and none of the ST121 genomes were positive for the *ars* operon (arsenic resistance) and for the *cadA4C* gene cassette (cadmium resistance). Further analyses on the localization of these two gene clusters within the genome, revealed that both the *ars* operon and the *cadA4C* gene cassette are located in a genomic region already identified as *Listeria* genomic Island 2 (LGI-2) (Lee et al., 2013; Parsons et al., 2017) (data not shown). In ST14 isolates included in the present study, LGI-2 was found in 24 out of 27 isolates and it was inserted within the *gtfA2* gene (data not shown). This gene codes for a sucrose phosphorylase involved in *O*-glycosylation of proteins. Glycosylation of flagellins is essential for bacterial flagellar assembly, motility, virulence, and host specificity (Merino and Tomás, 2014). The three isolates lacking LGI-2 (LSALM16, LSALM17, LSALM18) belonged to the persistent clone and were collected in September and November 2006 at the end of the sampling period. Further analyses should be performed in order to confirm whether these three isolates are natural mutants, which lost the accessory genome sequence of SGI-2.

Twenty *cadA4C* positive ST14 genomes additionally carried *cadA2C* gene cassette. The *cadA1C* gene cassette was found only in ST121 and in particular in 177 out of the 202 tested genomes (87.6%). Phenotypic tests conducted on the 33 *L. monocytogenes* strains from the rabbit meat-plant, confirmed that all *qacH* positive ST121 isolates were resistant to benzalkonium chloride and that all *cadA1C* positive ST121 isolates were resistant to 70 mg/l of cadmium chloride, whereas all *cadA4C* positive ST14 isolates were resistant to 35 mg/l of cadmium chloride.

As for environmental stress adaptation, no genomes were positive for the Stress Survival Islet 1 (SSI-1) and all ST121 but no ST14 were positive for the Stress Survival Islet 2 (SSI-2). All tested genomes carried a full-length version of genes associated to: cell-envelope stress response linked to bacteriocins resistance, adaptation to cold and/or high salt concentration, low pH, blue-light and desiccation.

Regarding biofilm formation, the *actA* gene was truncated in all ST121 and in none of the ST14 genomes. Phenotypic tests confirmed the differential biofilm forming ability of ST121 in comparison to ST14. In particular, tested ST121 isolates were classified as weak biofilm producers with ODC (OD Control) median value of 0.15 and OD values ranging from 0.17 to 0.24 OD, whereas all ST14 isolates but three (LSALM8, LSALM9, LSALM10) were categorized as moderate biofilm producers, with OD values ranging from 0.30 to 0.52 OD at 570 nm. OD values of ST121 isolates were statistically significant different in comparison to ST14 ones (*P* = 0,00238).

## Discussion

In the present study, the persistence of *L. monocytogenes* ST121 and ST14 repeatedly isolated within 1 year of sampling in a rabbit meat processing plant was studied by a genomic approach. Moreover, 94 putative gene markers of *L. monocytogenes* physiological adaptation to the food processing environment were investigated in newly sequenced as well as publicly available genomes of ST121 and ST14 strains. The aim was to evaluate the significant enrichment of these genes in the two subtypes, with a particular focus on those genes associated to ecophysiology in ST14, a subtype rarely isolated in food processing plants.

Based on PFGE-Typing, persistence of *L. monocytogenes* in dairy, meat, fish and vegetable sectors was extensively observed (Leong et al., 2014; Ortiz et al., 2015; Véghová et al., 2017). However, more recently, whole genome SNPs analysis revealed a superior discriminatory power. This is particularly relevant in studies in which highly similar strains have to be differentiated in order to distinguish true persistent from sporadic strains (Ferreira et al., 2014). SNPs calling revealed that the majority but not all of the ST14 isolates sharing the same PFGE-typing, belonged to the same clone. This clone, gathering 23 out of 27 isolates, was re-isolated more than six times within 1 year from rabbit carcasses, meat cuts, meat products and the processing environment in the same rabbit meat processing plant. Although an agreement has not been achieved on the definition of persistence, for the purpose of the present study, the ST14 clone was considered as persistent (Ferreira et al., 2014). This persistent clone included ST14 genomes sharing a maximum of 25 SNPs differences. A difference of 25 SNPs has been already proposed as cut off for definition of genetically related strains belonging to a single persistent clone of *L. monocytogenes* (Nielsen et al., 2017). The persistence of a *L. monocytogenes* strain for long periods confirms a higher risk of food contamination and human exposure to specific pathogen strains (Tompkin, 2002). The concern is even higher when the persistent clone belongs to subtype ST 14, which was described as including hypervirulent strains (e.g., ST14) (Mammina et al., 2013; Voronina et al., 2015; Maury et al., 2016; Palma et al., 2017).

Single nucleotide polymorphisms (SNPs) analysis gathered ST121 isolates in two clusters of 2 and 4 isolates each. SNPs analyses showed a higher discriminatory power in comparison to other molecular typing methods (*Apa*I-PFGE, 7-loci MLST, MLVA, ribotyping), all of which identified all ST121 isolates as belonging to the same clone (De Cesare et al., 2017). The analyzed dataset suggests that one ST121 clone gathering four isolates, survived over 5 months in the same rabbit meat processing plant. However, since those strains were repeatedly isolated in a relative short time frame, this clone cannot be considered as persistent.

In order to gain more insights on the genomic bases behind the differential frequency of ST121 and ST14 in food processing plants, a comprehensive literature review was performed in order to identify genes associated to physiological adaptation of *L. monocytogenes* to specific food-processing environmental stresses. In the literature, different dataset of genes associated to particular phenotypes, such as antimicrobial resistance and virulence of *L. monocytogenes*, have been described (Zankari et al., 2012; McArthur et al., 2013). However, to the best of author’s knowledges, no dataset on genes associated to ecophysiology of *L. monocytogenes* is available.

In the present study, 94 genes associated to ecophysiology were included in a dataset available for the public. Genes were included in the dataset only if their association to the specific phenotype was confirmed by insertional mutagenesis or deletion mutants experiments (**Supplementary Table S2**). In particular the dataset includes genes associated to resistance to antimicrobials, QAC, heavy metals and bacteriocins as well as associated to adaptation to cold, high salt concentration, low pH, desiccation and biofilm formation.

The results on 202 ST121 and 71 ST14 genomes outlined interesting findings with particular reference to genes significantly enriched in one or the other subtype (**Figure 3**). In particular genes *qacH*, *cadA1C*, Stress Survival Islet -2 and a truncated version of the *actA* gene were significantly enriched within the ST121 genomes (*P* < 0,000001), whereas the *ars* operon and *cadA4C* gene cassettes included in the *Listeria* Genomic Islands 2, the *bcrABC* locus and a full-length version of the *actA* gene were significantly enriched in ST14 genomes (*P* < 0,000001). The presence of a QAC associated genetic determinant (either the *qacH* gene or the *bcrABC* locus) was significantly enriched in ST121 genomes in comparison to ST14 (*P* < 0,000001). This observation underlines that subtypes ST121 and ST14 have different patterns of genes associated to ecophysiology. Further studies should confirm whether other subtypes with high and low frequency show the same pattern of genes as identified in ST121 and ST14 subtypes, respectively. Comparing the two patterns it appears that ST121 showed high adaptation to sanitizing procedures (resistance to QAC and adaptation to alkaline stress) (*qacH;* SSI-2) along with adaptation to high cadmium concentration (*cadA1C*) (Mullapudi et al., 2010; Müller et al., 2013; Harter et al., 2017). Moreover, ST121 harbored a truncated version of the *actA* gene whereas ST14 harbored a full-length version of the same gene. The *actA* gene is involved in the polymerization of actin which is important for motility of *L. monocytogenes* within the host cell as well as in the first steps of biofilm formation and in particular in cell-to-cell aggregation (Smith and Portnoy, 1997; Travier et al., 2013). Deletion mutants ΔactA showed attenuated virulence and inability to form biofilms (Travier et al., 2013). The higher biofilm forming ability of ST14 in comparison to ST121, was phenotypically confirmed.

All tested genomes carried a full-length version of genes associated to: cell-envelope stress response linked to bacteriocins resistance; adaptation to cold and/or high salt concentration, low pH and desiccation. These genes common to all *L. monocytogenes* genomes were not informative for the differentiation of the two ST121 and ST14 subtypes. For those genes, mostly located in the core genome, specific allele types can be identified. Genome wide association studies can be performed to associate specific allele types to specific phenotypes. Allele types with a strong association to the phenotype could be then included in the dataset. The usefulness of this approach was already demonstrated. For example a genome-wide association study (GWAS) on *Campylobacter jejuni*, revealed that, based on overrepresented genetic elements, different *C. jejuni* subtypes show distinct genotypes associated with survival from farm to fork (Yahara et al., 2017).

## Conclusion

The different gene enrichment found in ST121 and ST14 supported by phenotypic confirmations, suggest that ST121 mainly include strains resistant to sanitizers. This feature might in part explain the high frequency of detection of this subtype in food-processing plants. ST14 include biofilm producer strains. This feature suggest that ST14 might occasionally contaminate harborage sites where sanitizing procedures are difficult to be performed. If confirmed, this route of contamination might be of concern since ST14 could potentially spread from harborage sites to different food lots scattered in months or years. The concern is even higher if a hypervirulent strain is the driver of this repeated contamination.

## Author Contributions

FrP designed the study, performed the literature review of genes related to ecophysiology, and draft the manuscript. FeP performed data analysis and contributed in manuscript writing. LG contributed to the setup of the dataset. AL performed the phenotypic tests. ADC contributed to the literature review. GM collected the samples, revised the manuscript, and coordinated the study. All authors have contributed to data interpretation, have critically reviewed the manuscript, and approved the final version as submitted.

## Conflict of Interest Statement

The authors declare that the research was conducted in the absence of any commercial or financial relationships that could be construed as a potential conflict of interest.

## Footnotes

1 https://github.com/B-UMMI/INNUca

2 https://github.com/tseemann/snippy

3 https://github.com/rambaut/figtree

4 https://www.ncbi.nlm.nih.gov/pubmed/

5 https://blast.ncbi.nlm.nih.gov/Blast.cgi

6 https://www.ebi.ac.uk/Tools/msa/clustalo/

7 https://www.ncbi.nlm.nih.gov/nuccore/

8 https://github.com/tseemann/abricate

9 https://software.broadinstitute.org/morpheus/

## Supplementary Material

TABLE S1 Dataset of putative gene markers of ecophysiology in *L. monocytogenes*.

TABLE S2 Public genomes of *L. monocytogenes* ST121 and ST14 included in the present study.
